# Report of Secretary

**Published:** 1895-07

**Authors:** 


### SOCIETY OF HOMŒOPATHICIANS.

### MEETING OF 1895.

---

### FIRST DAY. MORNING SESSION.

---

ORIENTAL HOTEL, MANHATTAN BEACH, N. Y.

Wednesday, June 26th, 1895.

The first annual meeting of the Society of Homœopathicians was called to order by the Secretary at 3 P. M.

Dr. Edmund Carleton was elected Chairman.

The minutes of the last meeting for organization were read and approved.

*Report of Secretary, S. A. Kimball, M. D.*

Nothing of importance has occurred during the year with which you are not already familiar. It must be evident to all of us that we have made no mistake in organizing a new society and in establishing a home for pure Homœopathy. The necessity for it seems to be more and more apparent as time goes on. Our society will depend upon the efforts of its members for its usefulness, and if each of us will do his share of the work and present papers at each meeting for discussion there will be no question as to the success of our undertaking. I have nothing but routine business to report.

